# Author Correction: CDK2-mediated site-specific phosphorylation of EZH2 drives and maintains triple-negative breast cancer

**DOI:** 10.1038/s41467-020-14429-3

**Published:** 2020-01-29

**Authors:** Lei Nie, Yongkun Wei, Fei Zhang, Yi-Hsin Hsu, Li-Chuan Chan, Weiya Xia, Baozhen Ke, Cihui Zhu, Rong Deng, Jun Tang, Jun Yao, Yu-Yi Chu, Xixi Zhao, Ye Han, Junwei Hou, Longfei Huo, How-Wen Ko, Wan-Chi Lin, Hirohito Yamaguchi, Jung-Mao Hsu, Yi Yang, Dean N. Pan, Jennifer L. Hsu, Celina G. Kleer, Nancy E. Davidson, Gabriel N. Hortobagyi, Mien-Chie Hung

**Affiliations:** 1 Department of Molecular and Cellular Oncology, The University of Texas MD Anderson Cancer Center, Houston, TX 77030 USA; 2 Department of General Surgery, Xinhua Hospital affiliated to Shanghai Jiao Tong University School of Medicine, Shanghai, 20009 China; 3 Graduate School of Biomedical Sciences, The University of Texas Health Science Center at Houston, Houston, TX 77030 USA; 4 State Key Laboratory of Oncology in South China, Cancer Center, Sun Yat-Sen University, Guangzhou, China; 5 Department of Breast Oncology, Cancer Center, Sun Yat-Sen University, Guangzhou, China; 6 Department of Oncology, The Second Affiliated Hospital of Xi’an Jiaotong University, Shaanxi, China; 7 Department of Breast Surgery, Shengjing Hospital of China Medical University, Shenyyang, 110003 China; 8 Cancer Research Center, Qatar Biomedical Research Institute, Hamad Bin Khalifa University, Qatar Foundation, PO Box 34110, Doha, Qatar; 9 College of Liberal Arts, The University of Texas at Austin, Austin, TX 78712 USA; 10 Graduate Institute of Biomedical Sciences and Center for Molecular Medicine, and Office of the President, China Medical University, Taichung, 404 Taiwan; 11 Department of Biotechnology, Asia University, Taichung, 413 Taiwan; 12 Department of Pathology, University of Michigan Medical School, Ann Arbor, MI 48109 USA; 13 Fred Hutchinson Cancer Research Center, Seattle, WA 98109 USA; 14 Department of Breast Medical Oncology, The University of Texas MD Anderson Cancer Center Houston, Houston, TX 77030 USA

**Keywords:** Breast cancer, Cancer therapy, Targeted therapies

Correction to: *Nature Communications* 10.1038/s41467-019-13105-5, published online 8 November 2019.

The original version of this Article omitted the following funding source from the Acknowledgements:

CPRIT Research Training Program (RP101502, 140106, and 170067; to H.-W.K.). This has been corrected in the PDF and HTML versions of the Article.

