# Supplementary material for: CDK2-mediated site-specific phosphorylation of EZH2 drives and maintains triple-negative breast cancer
Source: Nat Commun. 2019 Nov 8;10:5114. doi: 10.1038/s41467-019-13105-5 (PMC6841924; doi:10.1038/s41467-019-13105-5)
Supplement: Supplementary file 1 — Supplementary Information [file 41467_2019_13105_MOESM1_ESM.pdf]

Supplementary Information

CDK2-mediated site-specific phosphorylation of EZH2 drives and maintains triple-negative breast cancer

Nie L et al.

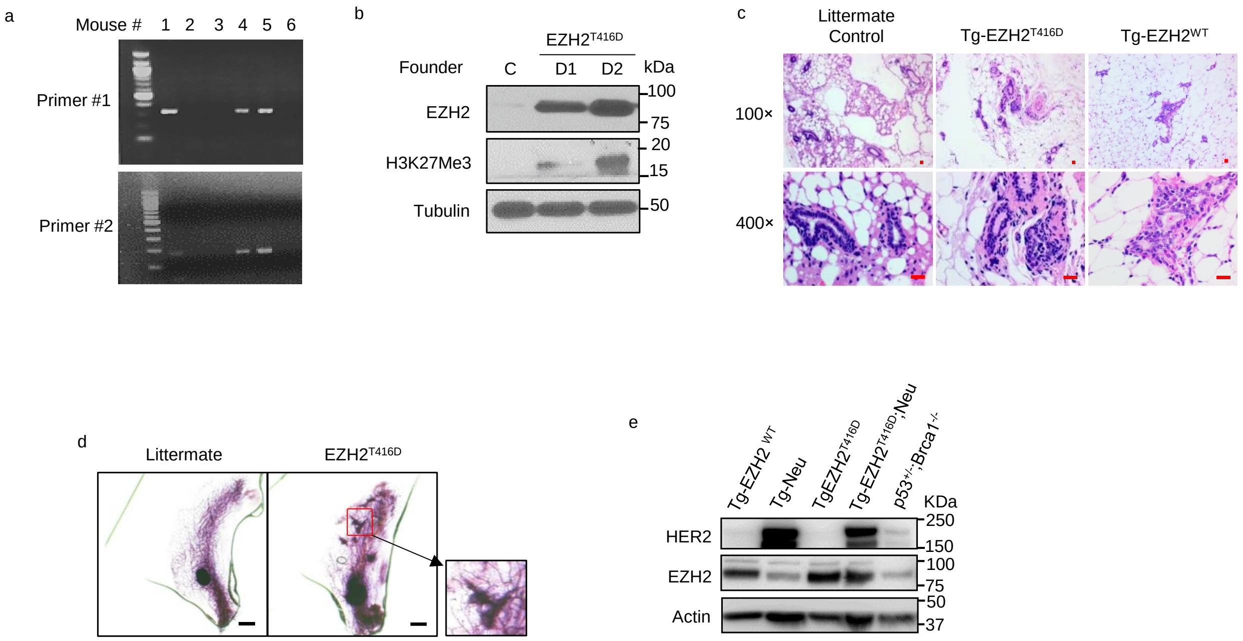

Supplementary Figure 1. Phenotypes of transgenic expression of EZH2^{T416D} mutant in mammary glands. (a) Genotyping of the transgene by PCR with two pairs of primers. 100bp DNA ladder (NEB) was used as DNA size marker. (b) Transgene EZH2 protein and histone3 K27-trimeythylation (H3K27Me3) levels in mammary epithelial cells of F1 female mice from two founders and a littermate. (c) *EZH2^{WT}* and *EZH2^{T416D}* transgenic mice driven by MMTV promoter were generated in FVB/N stain. Representative images of H&E staining of the mammary gland sections from 4-month old mice. Scale bar, 20 μm. (d) Representative images of the whole mount mammary gland staining. Scale bar, 2 mm. (e) Transgene EZH2 and HER2 expression levels in the mammary epithelial cells from the genetically engineering mouse lines with different genotypes.

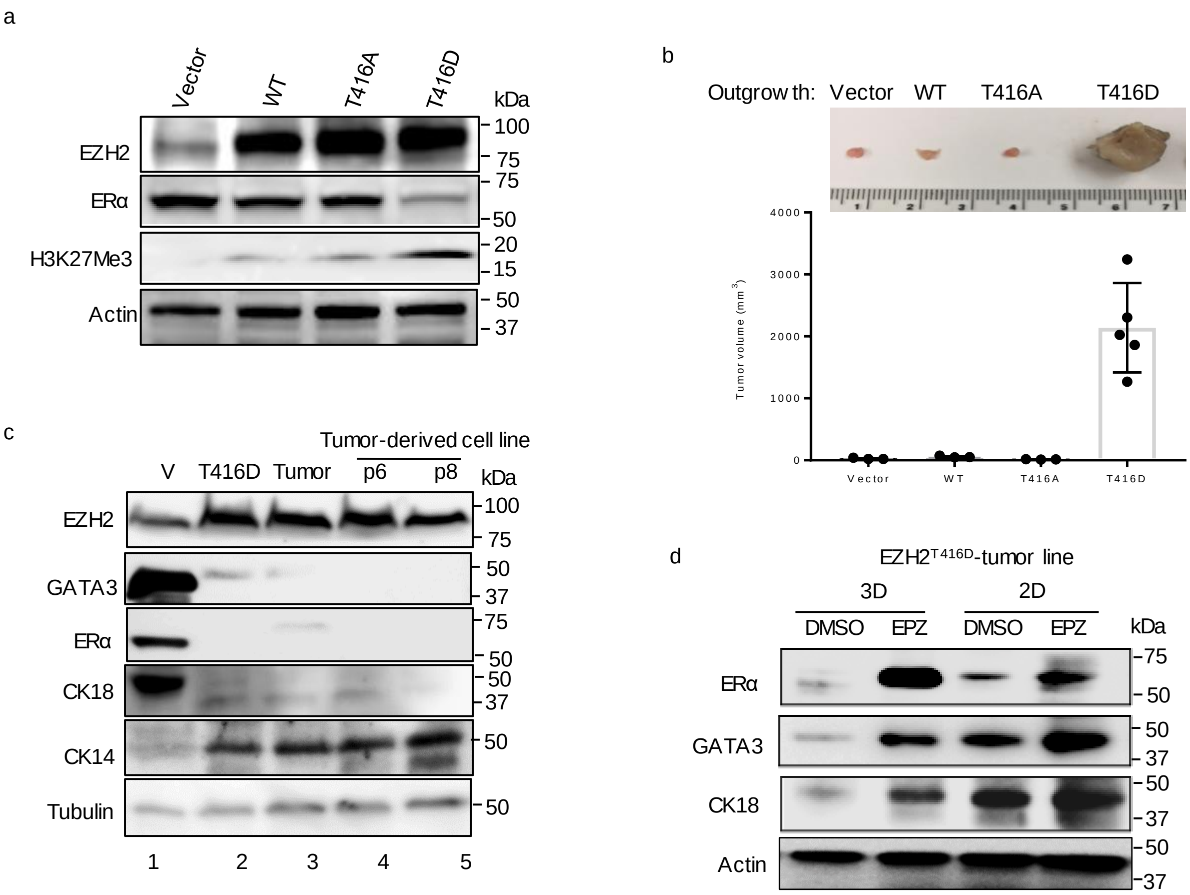

Supplementary Figure 2. The effects of EZH2^{T416D} expression in the luminal breast cancer T47D cells. (a) Ectopic expression of EZH2 T416 phospho-mimic T416D in luminal breast cancer cells downregulates ERα and upregulates H3K37Me3. (b) Expression of EZH2^{T416D} in non-tumorigenic breast cancer cells in NOD/SCID mice led to tumor development independent of estrogen. WT, EZH2^{WT}; T416A, EZH2^{T416A}; T416D, EZH2^{T416D}. Data in the bar graph were presented as *mean* ± *SD*. (c) Western blot analysis of luminal and basal-like markers in tumor-derived cell line and tumor tissue expressing EZH2^{T416D} in 3D Matrigel culture. (d) EZH2^{T416D} tumor–derived cell lines were isolated from the tumors and selected by short trypsinization followed by G418 selection. EZH2^{T416D}-derived cells were treated with EZH2 inhibitor EPZ-6438 (EPZ) at 25 µM for 72 hours and subjected to Western blot analysis with the indicated antibodies.

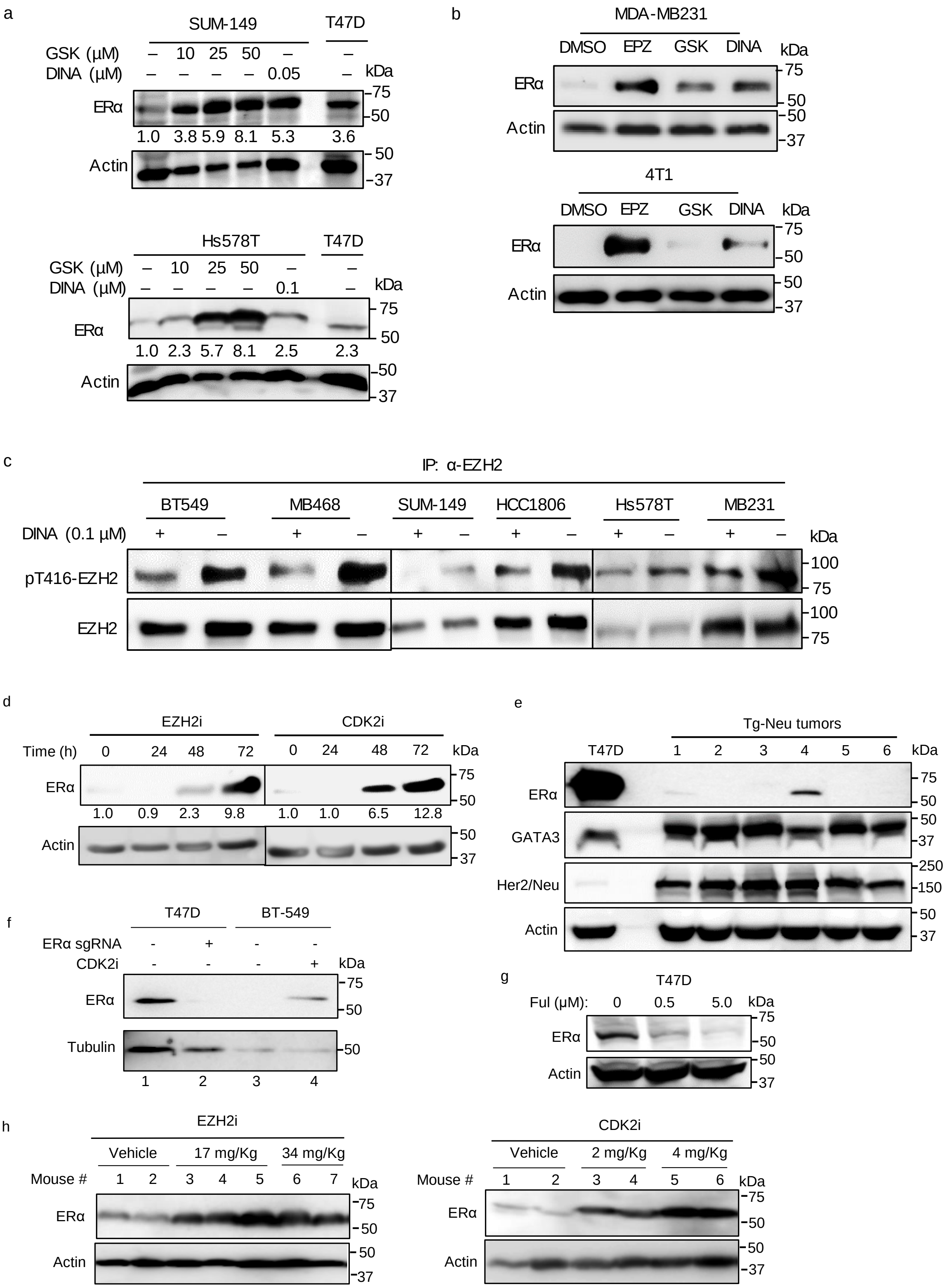
a
SUM-149
T47D
GSK (μM) – 10 25 50 – –
DINA (μM) – – – – 0.05 –
kDa
ERα
75
50
1.0 3.8 5.9 8.1 5.3 3.6
Actin
50
37
Hs578T
T47D
GSK (μM) – 10 25 50 – –
DINA (μM) – – – – 0.1 –
kDa
ERα
75
50
1.0 2.3 5.7 8.1 2.5 2.3
Actin
50
37
b
MDA-MB231
DMSO EPZ GSK DINA
kDa
ERα
75
50
Actin
50
37
4T1
DMSO EPZ GSK DINA
kDa
ERα
75
50
Actin
50
37
c
IP: α-EZH2
BT549 MB468 SUM-149 HCC1806 Hs578T MB231
DINA (0.1 μM) + – + – + – + – + – + –
kDa
pT416-EZH2
100
75
EZH2
100
75
d
EZH2i
CDK2i
Time (h) 0 24 48 72 0 24 48 72
kDa
ERα
75
50
1.0 0.9 2.3 9.8 1.0 1.0 6.5 12.8
Actin
50
37
e
T47D
Tg-Neu tumors
1 2 3 4 5 6
kDa
ERα
75
50
GATA3
50
37
Her2/Neu
250
150
Actin
50
37
f
T47D
BT-549
ERα sgRNA - + - -
CDK2i - - - +
kDa
ERα
75
50
Tubulin
50
1 2 3 4
g
T47D
Ful (μM): 0 0.5 5.0
kDa
ERα
75
50
Actin
50
37
h
EZH2i
Vehicle
17 mg/Kg
34 mg/Kg
Mouse # 1 2 3 4 5 6 7
kDa
ERα
75
50
Actin
50
37
CDK2i
Vehicle
2 mg/Kg
4 mg/Kg
Mouse # 1 2 3 4 5 6
kDa
ERα
75
50
Actin
50
37

Supplementary Figure 3. Inhibition of CDK2/EZH2 axis in TNBC cells reactivates ERα expression. (a) SUM-149 (top) and Hs578T (bottom) cells were treated with increasing concentrations of EZH2i (GSK) or CDK2i (DINA) for 72 hours. Cell lysates were harvested and subjected to Western blotting with the indicated antibodies. Quantitation analyses of the images were performed by using ImageQuant TL Toolbox v8.1 and normalized with loading control actin. (b) Human MDA-MB-231 and murine 4T1 cells were treated with EZH2i (EPZ and GSK) or CDK2i (DINA) followed by Western blotting with the indicated antibodies. Actin was used as loading control. (c) The indicated cells were treated with CDK2i (DINA) and subjected to immunoprecipitation by EZH2 antibody followed by Western blotting with the monoclonal antibody against pT416-EZH2. (d) Time-course of ERα expression induction by EZH2i (GSK) or CDK2i (DINA). MDA-MB231 cells were treated with EZH2i (GSK; 12.5 μM) or CDK2i (DINA; 50 nM) for the indicated times and subjected to Western blot analysis for ERα protein level with an ERα antibody. Quantitation analyses of the images was performed as described in (a). (e) Protein levels of GATA3 and ERα in the mammary gland tumors from Tg-Neu mice. ERα, GATA3 and transgene Her2/Neu proteins in whole lysates of six primary tumor tissues wer e immuno-blotted with indicated antibodies. Luminal breast cancer cell line T47D was used for control (f) Anti-ERα antibody specifically recognized ERα in ERα$^+$ T47D cells and CDK2i-induced BT-549 cells. Cas-9/sgRNA targeting ERα (Thermo Fisher) in T47D cells abolished ERα signal in western blot analysis. (g) ERα in the luminal cell line T47D is decreased with increased fulvestrant. ERα protein was immunoblotted with anti-ERα from Abcam. (h) Balb/c female mice were inoculated with 4T1 tumor cells and treated with indicated dose of EZH2i (EPZ), CDK2i (DINA), or vehicle for 3 days. Tumor tissues were harvested, and lysates were subjected to Western blotting with specific ERα antibody. Actin was used as loading control. DINA, dinaciclib. EPZ, EPZ-6438. GSK, GSK343.

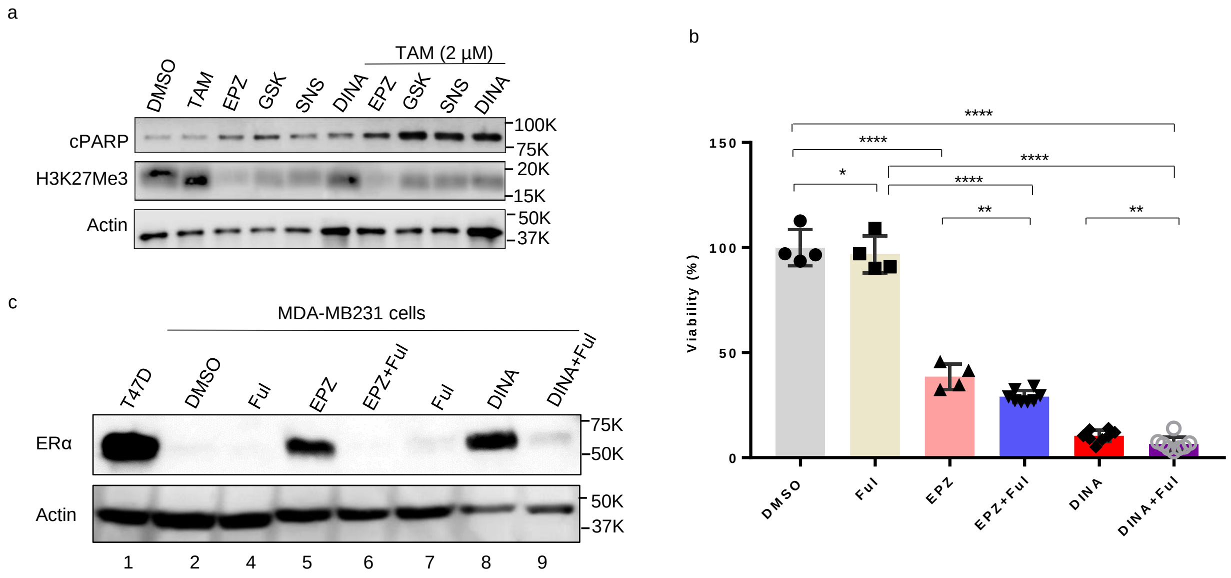

Supplementary Figure 4. Synergistic inhibitory effect of CDK2i/EZH2i combined with hormone therapy. (a) Combination treatment of the TNBCs in 3D culture with CDK2/EZH2 inhibitors and TAM induces apoptosis. An increased cleavage PARP protein (cPARP) in the combined treatment was detected by immunoblot. H3K27Me3 level was used as an index for inhibitory efficacy of the inhibitors. (b) Cell viability was determined by MTT assy. TNBC cells were treated with CDK2i or EZH2i combined with fulvestrant. Data were presented as *mean* ± *SD* n = 3. **** $p < 0.001$, ** $p < 0.05$, *not significant. Student's T test. (c) Western blot analysis of ERα protein levels in the cells treated with or without the indicated drug treatment using an a specific ERα antibody. DINA, dinaciclib. EPZ, EPZ-6438. Ful, fulvestrant.

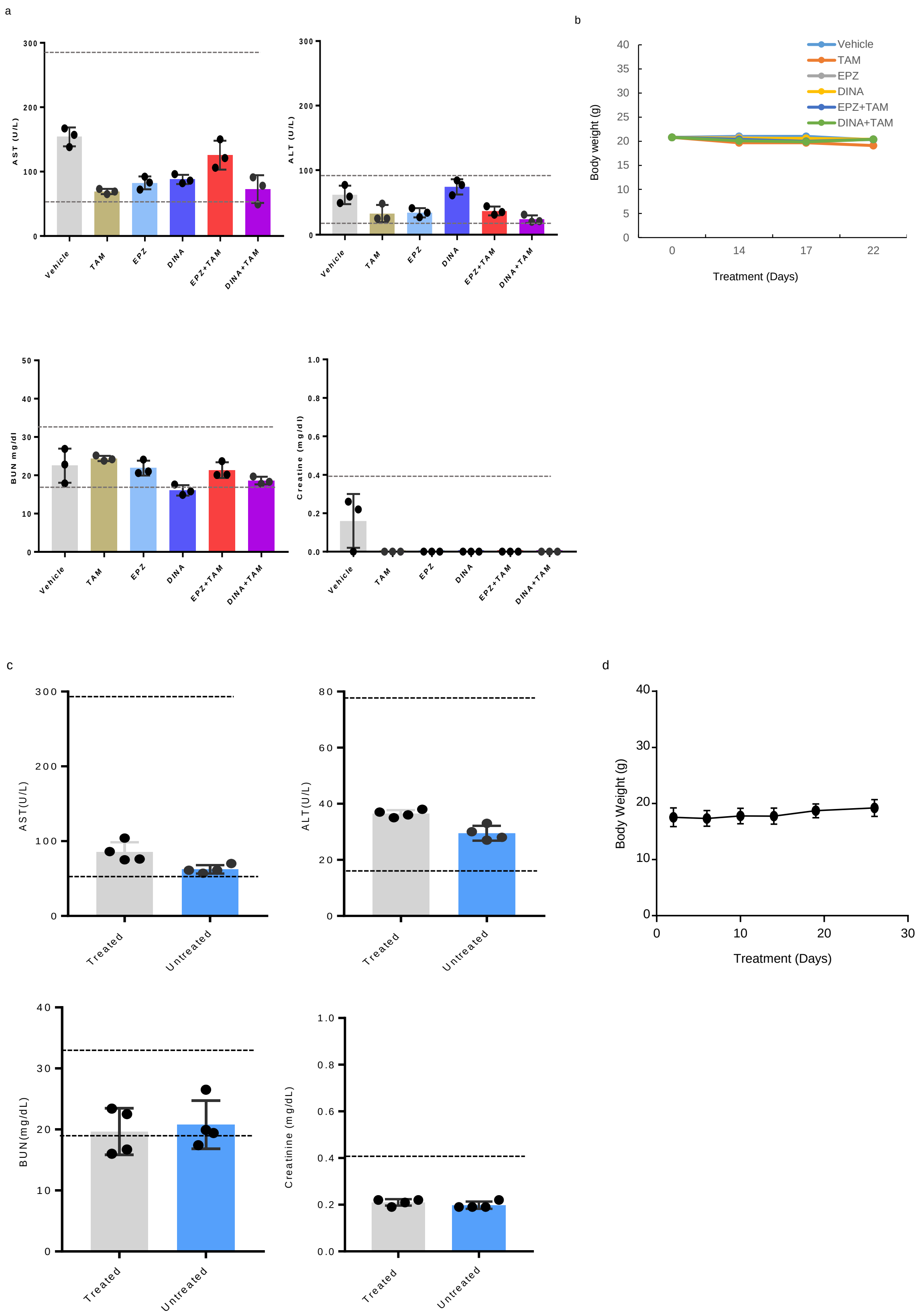
a
AST (U/L)
ALT (U/L)
BUN mg/dl
Creatine (mg/dl)
Vehicle
TAM
EPZ
DINA
EPZ+TAM
DINA+TAM
b
Body weight (g)
Treatment (Days)
Vehicle
TAM
EPZ
DINA
EPZ+TAM
DINA+TAM
c
AST(U/L)
ALT(U/L)
BUN(mg/dL)
Creatinine (mg/dL)
Treated
Untreated
d
Body Weight (g)
Treatment (Days)

Supplementary Figure 5. Toxicity of combination therapy of CDK2i or EZH2i and TAM *in vivo*. (a and b) Changes in liver and kidney function indicators, and body weight of mice from Figure 4 (c and d) after administration of EZH2i (EPZ-6438; 34 mg kg^-1) or CDK2i (DINA; 8 mg kg^-1) alone or in combination with tamoxifen (4 mg kg^-1) for 15 days. (c) Changes in liver and kidney function indicators, and body weight of mice administered the combination (+) of CDK2i (8 mg kg^-1) and tamoxifen (4 mg kg^-1) for 21 days compared with those without (–) treatment (n = 4). Dashed lines indicate the normal range. AST, aspartate aminotransferase. ALT, alanine aminotransferase. BUN, Blood urea nitrogen.

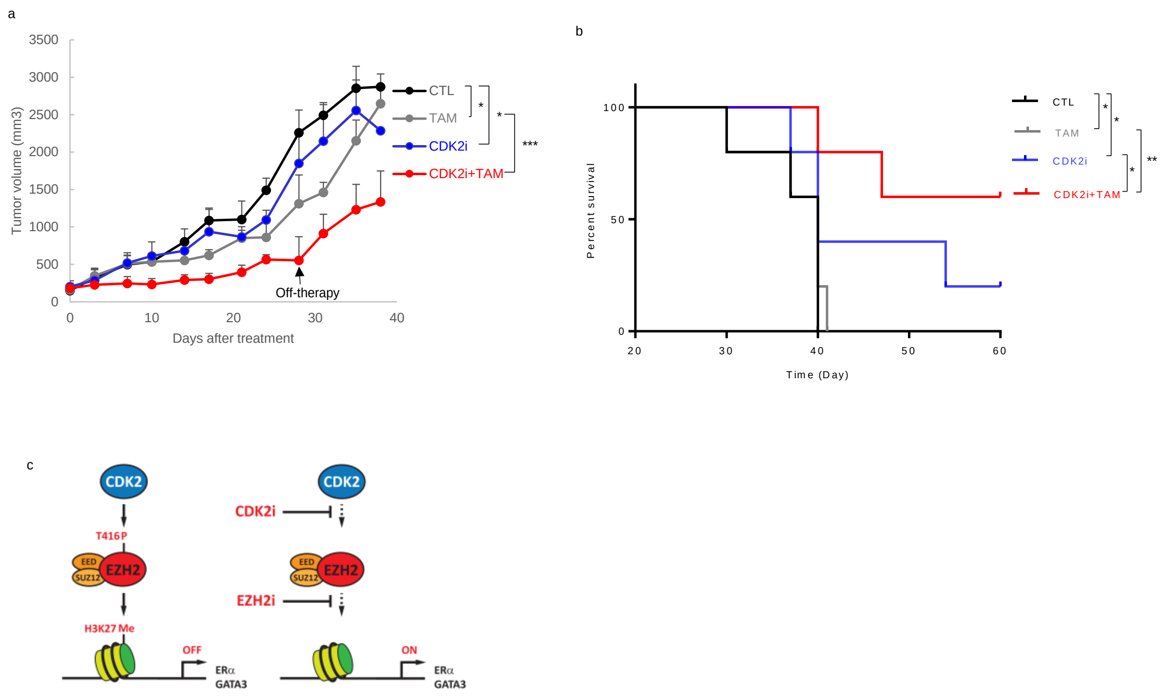

Supplementary Figure 6. CDK2i at low dose combined with tamoxifen improve the survival of host mice. (a) The inhibitory effects of low dose of CDK2i (dinaciclib) in combination with tamoxifen (TAM) compared with CDK2i alone, TAM alone, and control (vehicle) in MDA-MB-231 breast cancer xenograft tumor model. MDA-MB-231 cells (1×10^6) mixed with equal volume of Matrigel were orthotopically inoculated into the mammary fat pad of nude mice. When tumor size reached 160 mm^3, mice were treated with non-DLT regimen of CDK2i (2 mg kg^-1) plus TAM (4 mg kg^-1) for 4 weeks. Arrow indicates the date when therapy stopped. *** $p < 0.01$, *not significant. Student's t test. n = 5. (b) Survival curves of the non-DLT combination in TNBC tumor-bearing mice treated as described above (a) and monitored for two months. ** $p < 0.05$, *not significant. n = 5. Statistical analysis of survival rate was performed in Graph Pad Prism 7 software (Log-Rank test). (c). Working model. (Left) CDK2-mediated specific site T416 phosphorylation is required for guiding the PRC2 complex to bind to the lineage-specific promoters such as ERα and GATA3. pT416-EZH2 containing PRC2 complex biasedly silences ERα and GATA3 expression; (Right) Blockade of CDK2/EZH2 signaling axis by inhibitors reactivates ERα and luminal marker gene expressions.

Supplementary Table 1 Antibodies used in the study

Antibody	Catalog No.	Vendor	Experiments	Dilutions
Actin	A2066	Sigma-Aldrich	WB	1:1000
CDK2	Ab32147	Abcam	WB	1:1000
CK14	Ab181595	Abcam	IHC, WB	1:100, 1:1000
CK18	Bs-2043R	Bioss	IHC, WB	1:100, 1:1000
Cleaved PARP-1	Sc-56196	Santa Cruz	WB	1:1000
E-cadherin	#610405	BD Transduction Laboratory	WB	1:1000
EpCAM	Ab32392	Abcam	WB	1:1000
Erb2/Her2	MA5-13105	Thermo Fisher		1:1000
EZH2	#61045	BD Transduction Laboratory	ChIP, IP	5 µg
EZH2	#5246	Cell Signaling Technology	WB	1:1000
p-T416EZH2		Generated in the laboratory	WB	1:200 (Ascites)
GATA3	#653802	BioLegend	WB	1:1000
H3K27Me3	#07-449	EMD Millipore	WB, ChIP	1:3000, 5 µg
Phopho-CDK2 (Thr 160)	#2561	Cell Signaling Technology	WB	1:1000
Tubulin	T9026	Sigma-Aldrich	WB	1:2000

WB, Western blotting; IHC, immunohistochemistry; ChIP, chromatin immunoprecipitation; IP, immunoprecipitation
