# Supplementary material for: CDK2-mediated site-specific phosphorylation of EZH2 drives and maintains triple-negative breast cancer
Source: Nat Commun. 2019 Nov 8;10:5114. doi: 10.1038/s41467-019-13105-5 (PMC6841924; doi:10.1038/s41467-019-13105-5)
Supplement: Supplementary file 3 — Description of Additional Supplementary Files [file 41467_2019_13105_MOESM3_ESM.pdf]

Description of Additional Supplementary Files

File Name: Supplementary Data 1

Description: RNAseq was performed by NOVOGENE (UC Davis) and Homo sapiens (GRCH37/hg19) was used as reference genome annotation. R package limma was used to identify upregulated and down-regulated genes after EZH2i and CDK2i treatment, using threshold cutoffs of p value 0.05, fold change above 2, and genes with higher expression having a log2 FPKM value above 3. The intersection of EZH2i and CDK2i altered gene expression identified 108 genes, of which 46 were up after inhibitor treatment and 62 down after inhibitor treatment. We used DAVID to annotate genes for biological functions and pathways (https://david.ncifcrf.gov)
