# Supplementary material for: CDK2-mediated site-specific phosphorylation of EZH2 drives and maintains triple-negative breast cancer
Source: Nat Commun. 2019 Nov 8;10:5114. doi: 10.1038/s41467-019-13105-5 (PMC6841924; doi:10.1038/s41467-019-13105-5)
Supplement: Supplementary file 5 — Reporting Summary [file 41467_2019_13105_MOESM5_ESM.pdf]

nature research

Corresponding author(s): Dr. Mien-Chie Hung

Reporting Summary

Nature Research wishes to improve the reproducibility of the work that we publish. This form provides structure for consistency and transparency in reporting. For further information on Nature Research policies, see Authors & Referees and the Editorial Policy Checklist.

Statistical parameters

When statistical analyses are reported, confirm that the following items are present in the relevant location (e.g. figure legend, table legend, main text, or Methods section).

n/a	Confirmed	
☐	☒	The exact sample size (n) for each experimental group/condition, given as a discrete number and unit of measurement
☐	☒	An indication of whether measurements were taken from distinct samples or whether the same sample was measured repeatedly
☐	☒	The statistical test(s) used AND whether they are one- or two-sided<br>*Only common tests should be described solely by name; describe more complex techniques in the Methods section.*
☒	☐	A description of all covariates tested
☒	☐	A description of any assumptions or corrections, such as tests of normality and adjustment for multiple comparisons
☐	☒	A full description of the statistics including central tendency (e.g. means) or other basic estimates (e.g. regression coefficient) AND variation (e.g. standard deviation) or associated estimates of uncertainty (e.g. confidence intervals)
☐	☒	For null hypothesis testing, the test statistic (e.g. F, t, r) with confidence intervals, effect sizes, degrees of freedom and P value noted<br>*Give P values as exact values whenever suitable.*
☒	☐	For Bayesian analysis, information on the choice of priors and Markov chain Monte Carlo settings
☒	☐	For hierarchical and complex designs, identification of the appropriate level for tests and full reporting of outcomes
☒	☐	Estimates of effect sizes (e.g. Cohen's d, Pearson's r), indicating how they were calculated
☐	☒	Clearly defined error bars<br>*State explicitly what error bars represent (e.g. SD, SE, CI)*

Our web collection on statistics for biologists may be useful.

Software and code

Policy information about availability of computer code

Data collection	Illumina NovaSeq 6000,HiSeq 4000 (Illumina Platform PE150)
Data analysis	GraphPad Prism, SPSS, Excel, MEDCALC

For manuscripts utilizing custom algorithms or software that are central to the research but not yet described in published literature, software must be made available to editors/reviewers upon request. We strongly encourage code deposition in a community repository (e.g. GitHub). See the Nature Research guidelines for submitting code & software for further information.

Data

Policy information about availability of data

All manuscripts must include a data availability statement. This statement should provide the following information, where applicable:

- Accession codes, unique identifiers, or web links for publicly available datasets
- A list of figures that have associated raw data
- A description of any restrictions on data availability

data for figures are provided in the paper. RNAseq data is available in The NCBI GEO repository with an accession ID GSE132194.

Field-specific reporting

Please select the best fit for your research. If you are not sure, read the appropriate sections before making your selection.

☒ Life sciences ☐ Behavioural & social sciences ☐ Ecological, evolutionary & environmental sciences

For a reference copy of the document with all sections, see nature.com/authors/policies/ReportingSummary-flat.pdf

Life sciences study design

All studies must disclose on these points even when the disclosure is negative.

Sample size	No Statistical method was used to predetermine sample size.
Data exclusions	N/A
Replication	Our experimental findings were confirmed with at least 3 times independent experiments
Randomization	The experiment were not randomized.
Blinding	IHC analysis was performed without any information about patient tissue.<br>Blinding was not used for animal works.

Reporting for specific materials, systems and methods

Materials & experimental systems

n/a	Involved in the study
☒	☐ Unique biological materials
☐	☒ Antibodies
☐	☒ Eukaryotic cell lines
☒	☐ Palaeontology
☐	☒ Animals and other organisms
☒	☐ Human research participants

Methods

n/a	Involved in the study
☒	☐ ChIP-seq
☒	☐ Flow cytometry
☒	☐ MRI-based neuroimaging

Antibodies

Antibodies used	We provide the information in Online Methods
Validation	all of the antibody was validated by manufacturer or us

Eukaryotic cell lines

Policy information about cell lines

Cell line source(s)	American Type Culture Collection (ATCC)
Authentication	Cells were authenticated by short tandem repeat DNA finger printing.
Mycoplasma contamination	they were negative for mycroplasma
Commonly misidentified lines<br>(See ICLAC register)	NO

Animals and other organisms

Policy information about studies involving animals; ARRIVE guidelines recommended for reporting animal research

Laboratory animals	J/Nu (007850), FVB/NJ (001800), C57BL/6J (000664) and Balb/c (000651) female mice were purchased from The Jackson

Laboratory animals	Laboratory
Wild animals	N/A
Field-collected samples	N/A
