# Supplementary material for: CDK2-mediated site-specific phosphorylation of EZH2 drives and maintains triple-negative breast cancer
Source: Nat Commun. 2019 Nov 8;10:5114. doi: 10.1038/s41467-019-13105-5 (PMC6841924; doi:10.1038/s41467-019-13105-5)

Fig. 1e

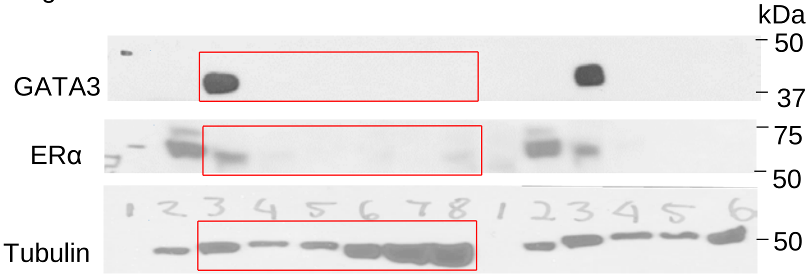


Fig. 1f

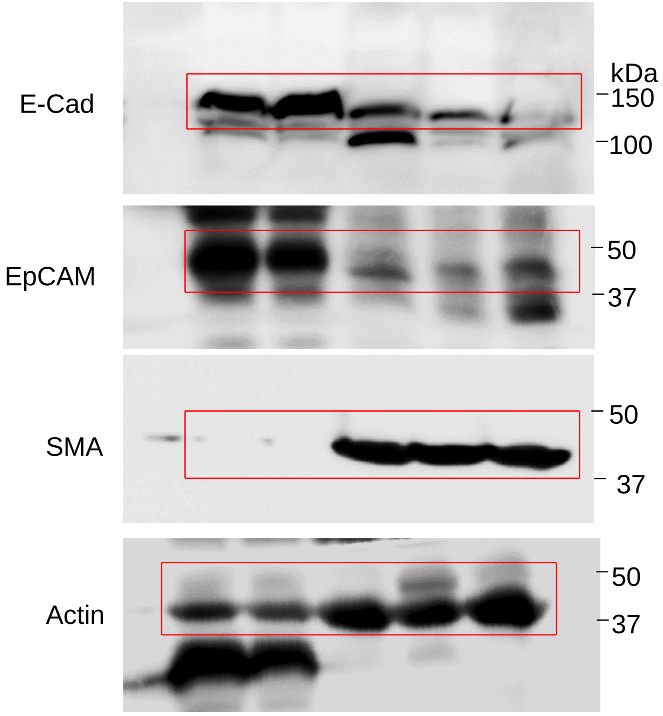


Fig.1h

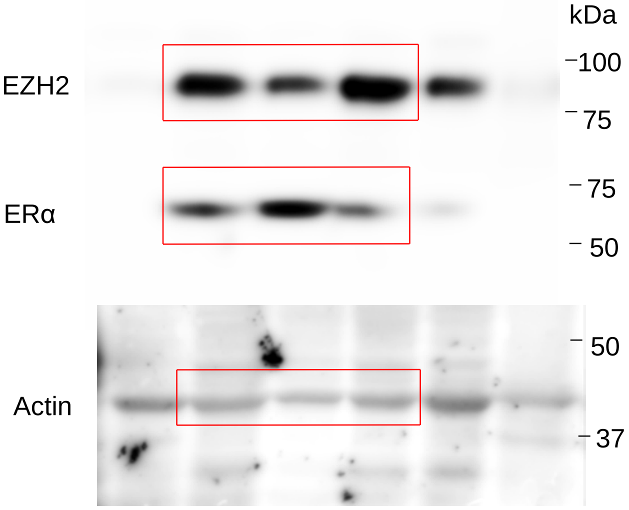

Fig. 2b

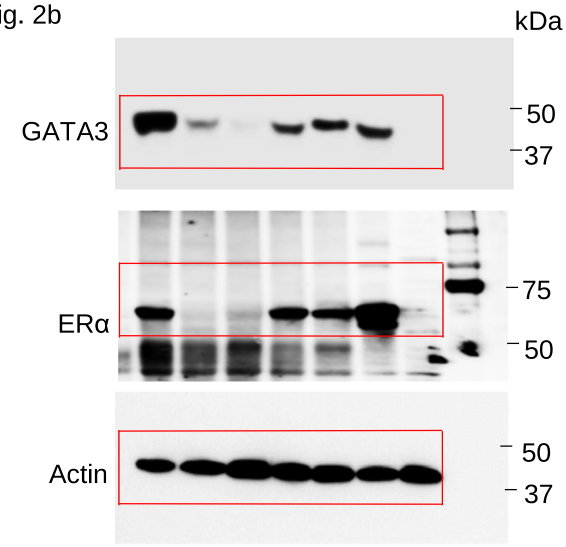


Fig. 2c

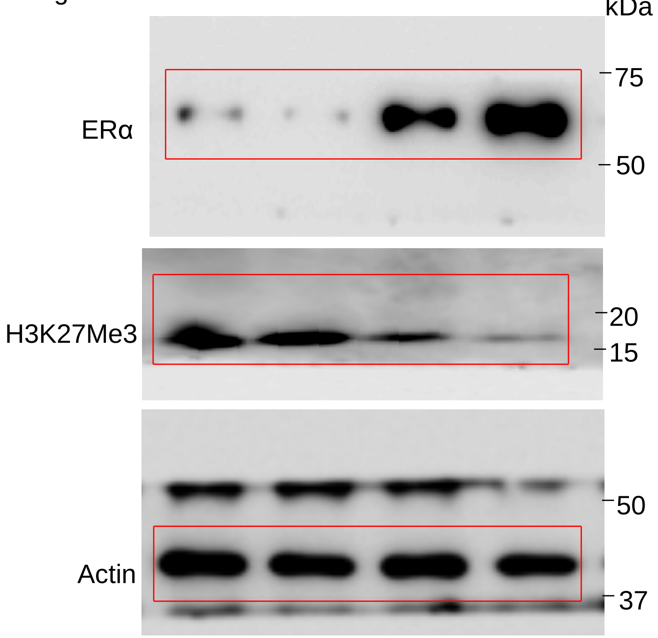


Fig. 2d

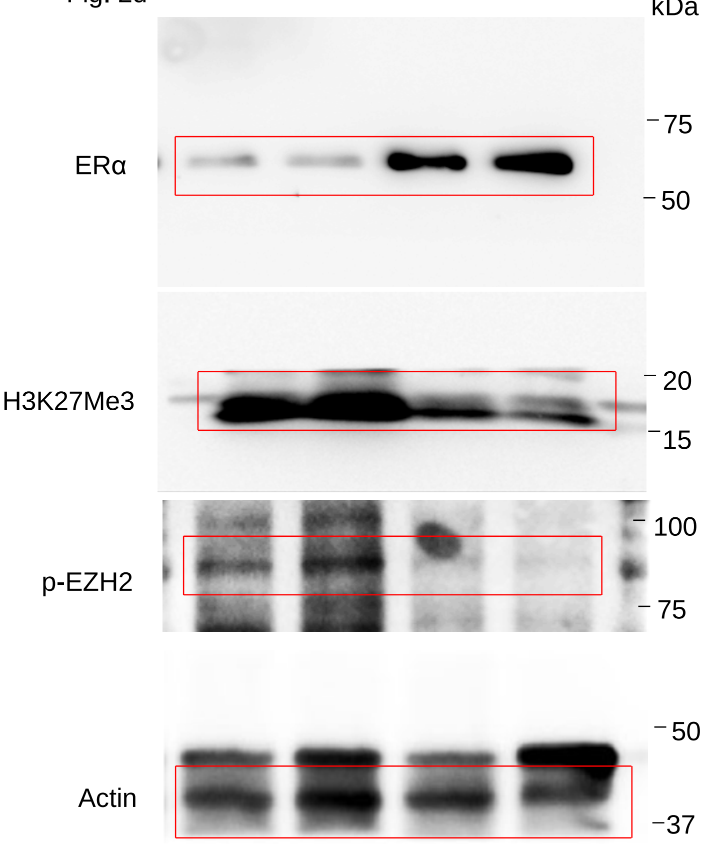


Fig. 2e

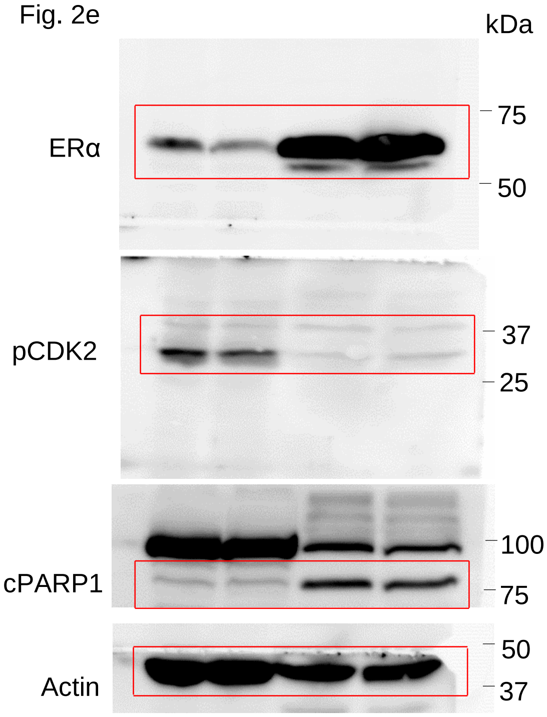

Fig. 2f

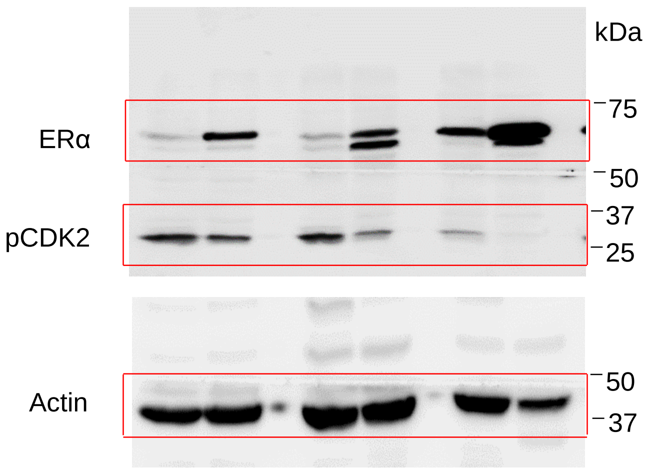


Figure 2h

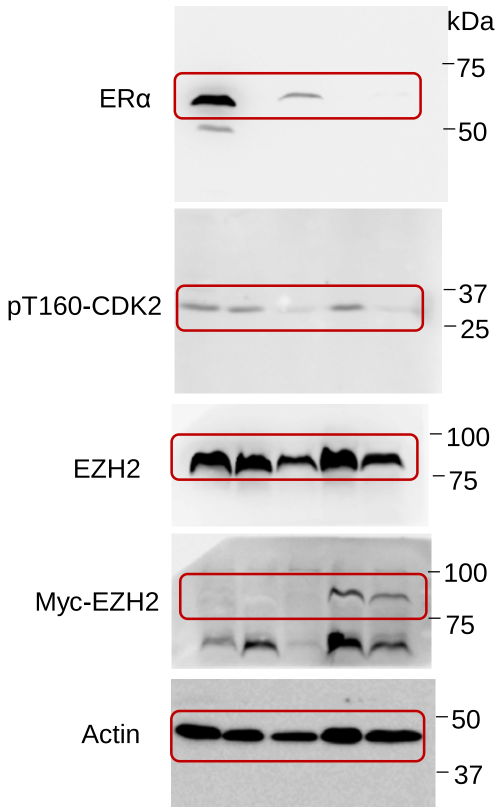


Fig. 2g

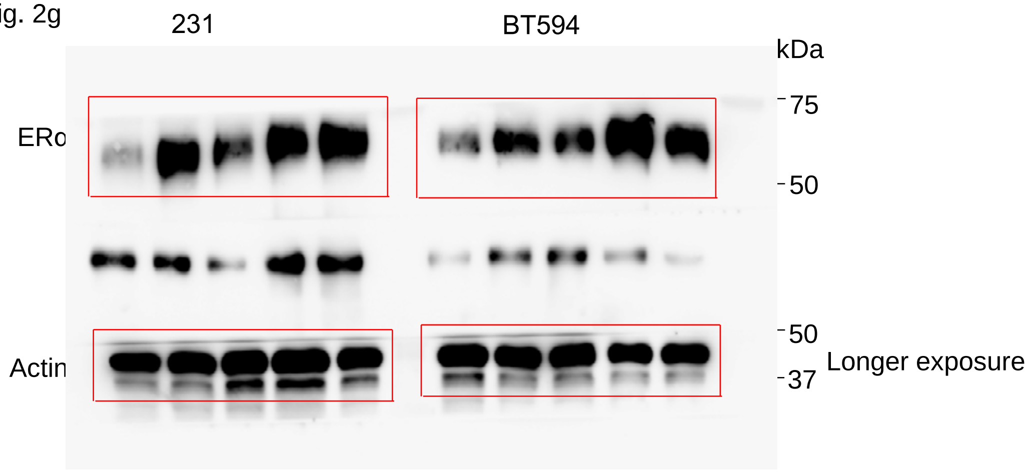

Suppl. Fig. 1b

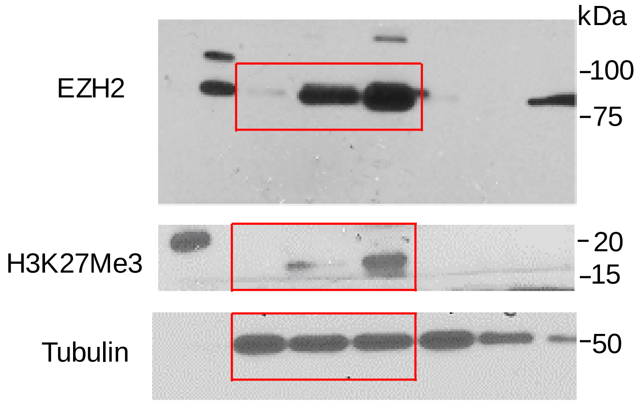


Suppl. Fig. 1e

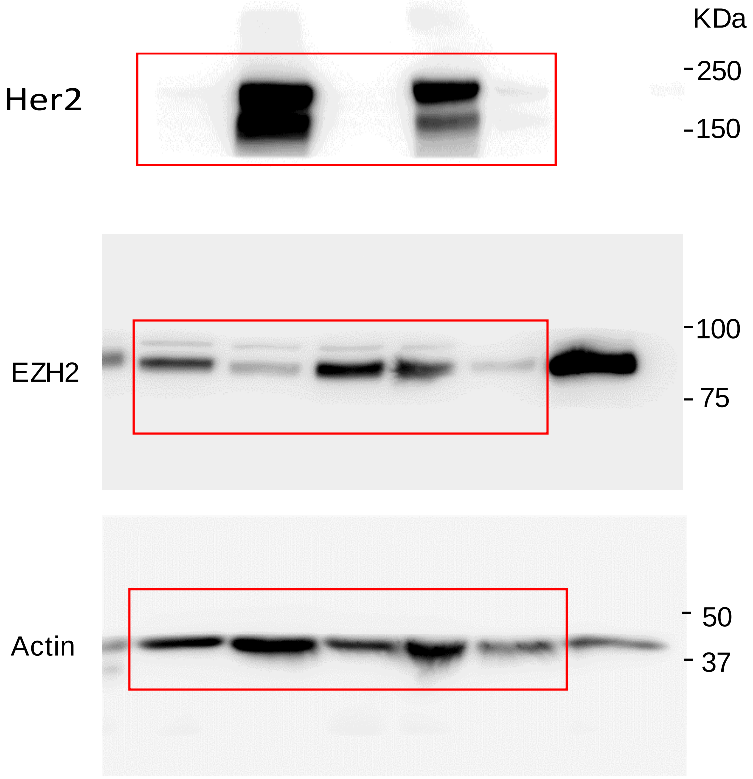

Suppl. Fig. 2a

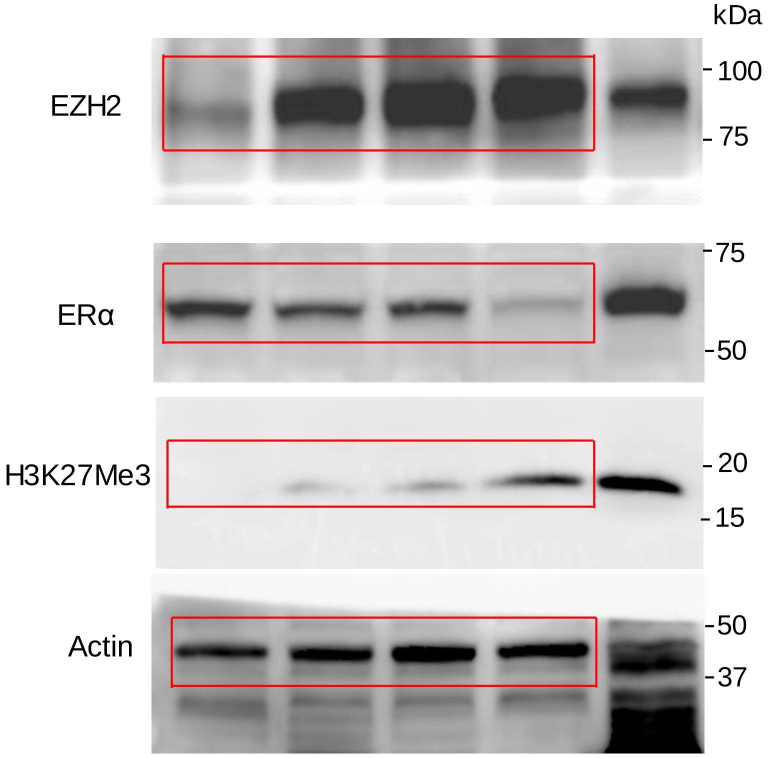


Suppl .Fig. 2c

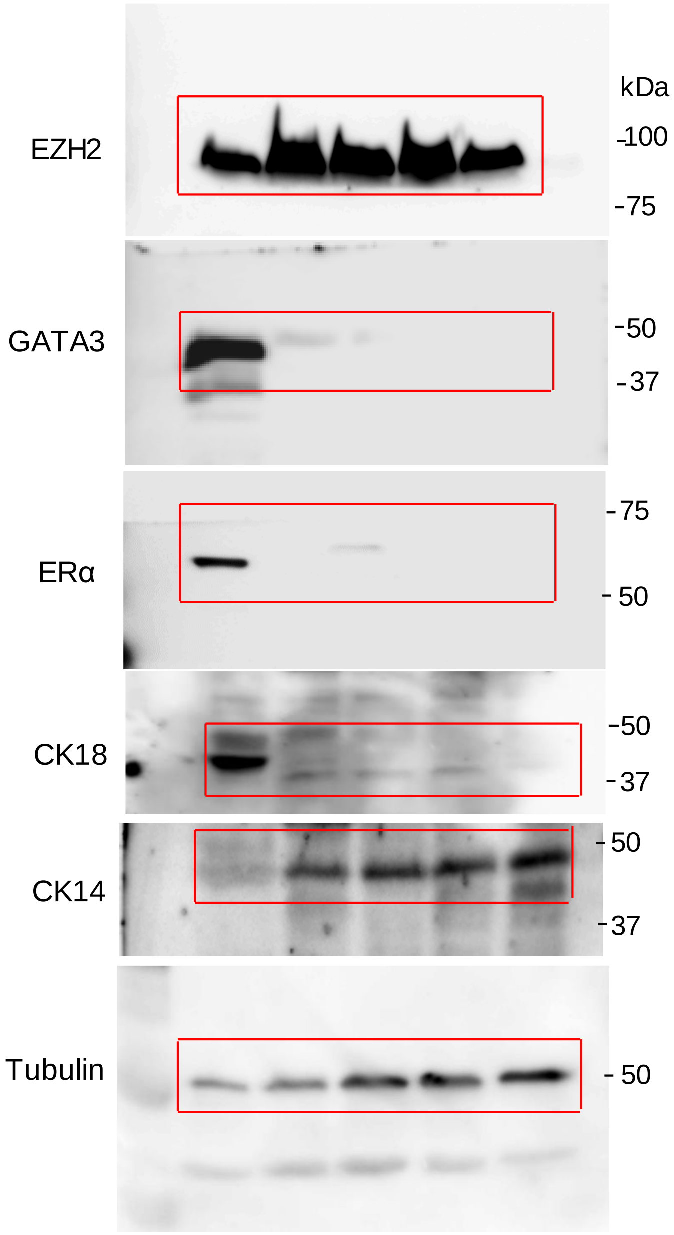


Suppl. Fig. 2d

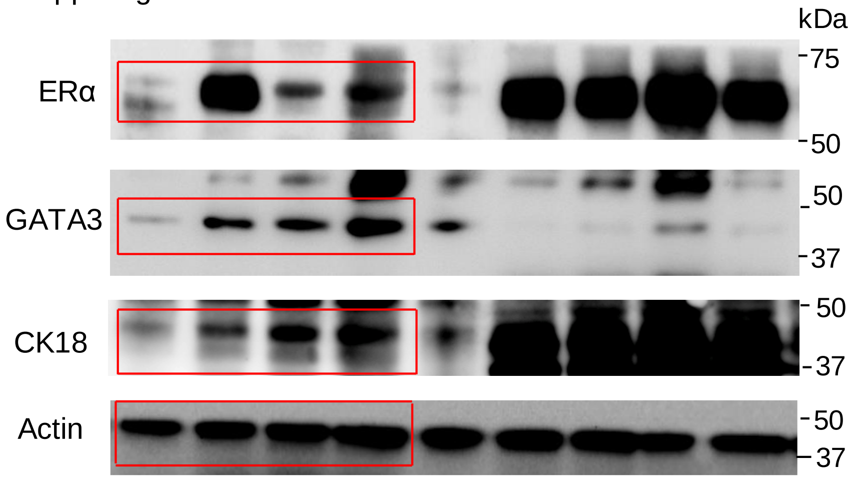

Suppl. Fig. 3a

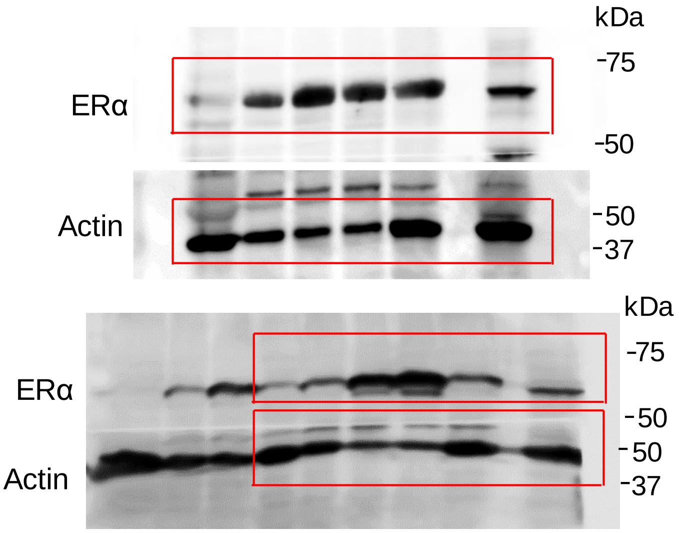


Suppl. Fig. 3b

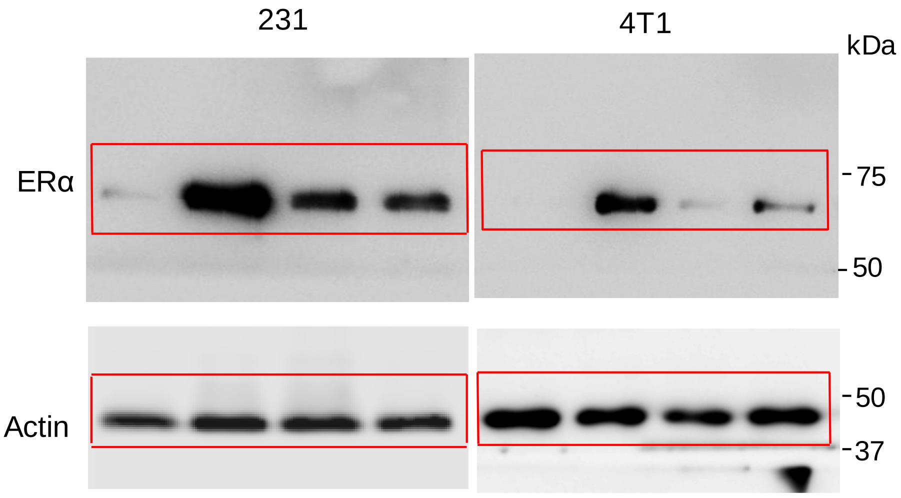


Suppl. Fig. 3c

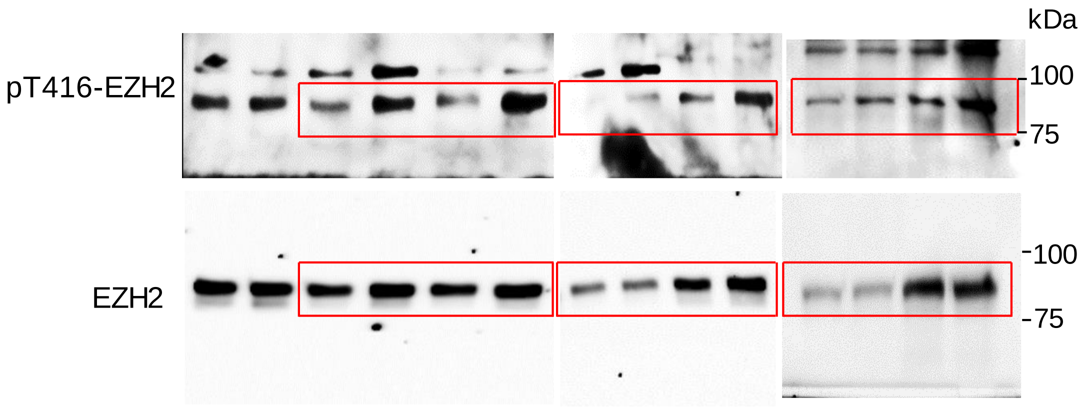


Suppl. Fig. 3d

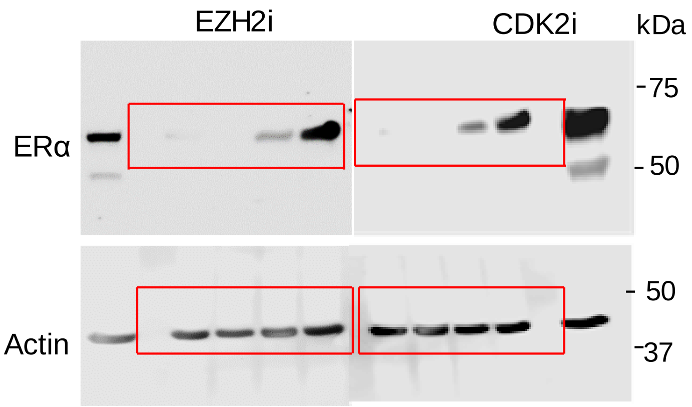

Suppl. Fig. 3e

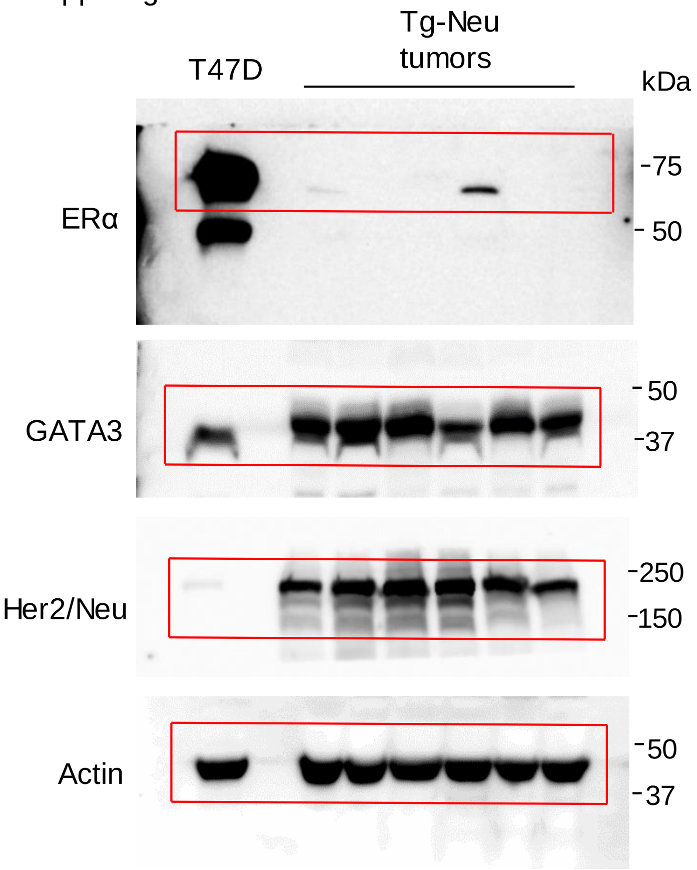


Suppl. Fig 3f

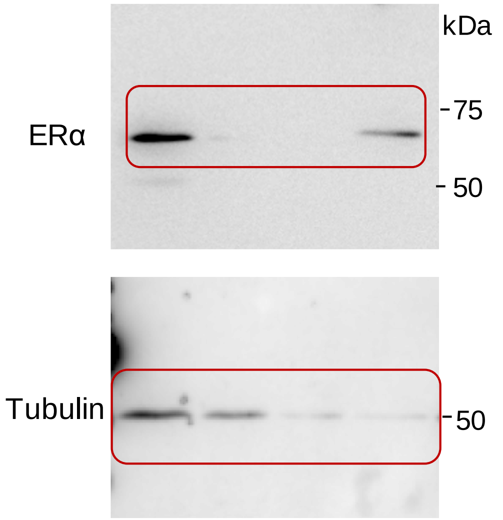


Suppl. Fig. 3g

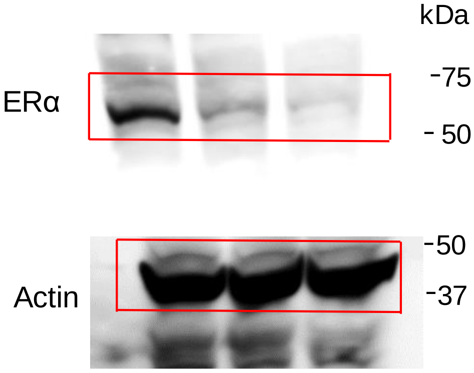


Suppl. Fig. 3h

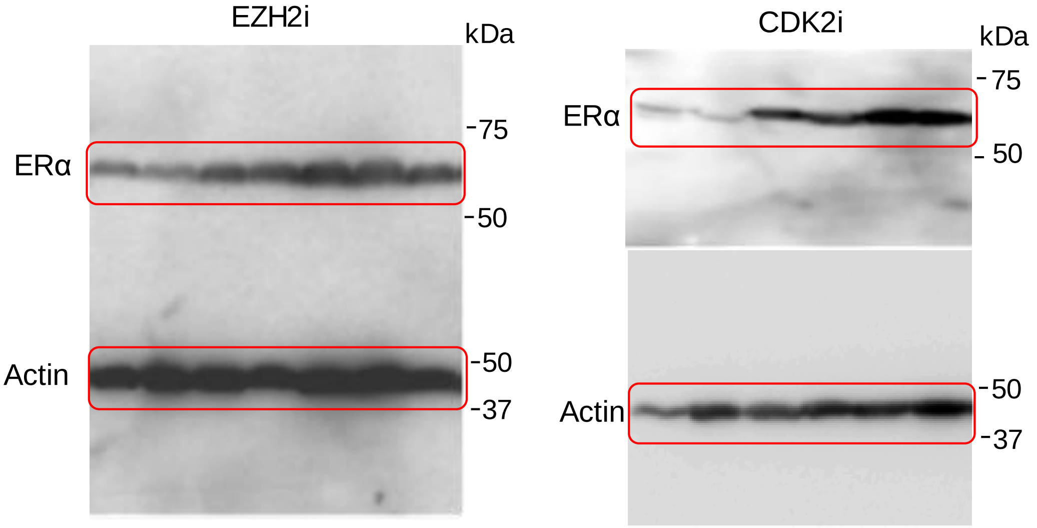

Suppl. Fig.4a

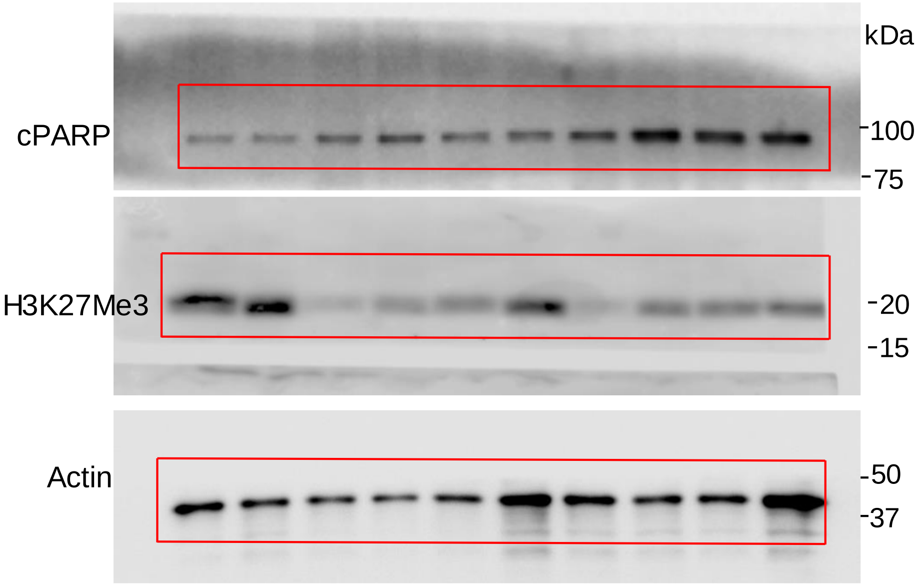


Suppl. Fig. 4c

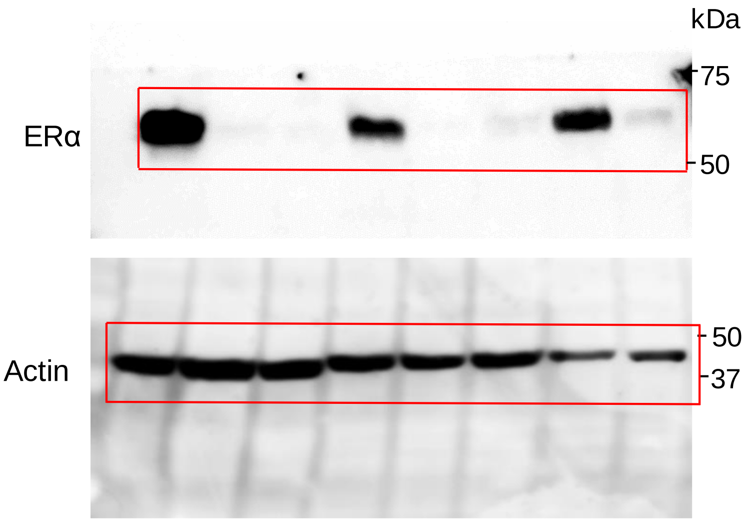

Supplement: Supplementary file 6 — Source Data [file 41467_2019_13105_MOESM6_ESM.zip › Source Data Files/Source Data File - Blots.pdf]
